# Safety and efficacy of inferior vena cava filter retrieval: a 5-year single center retrospective review from a tertiary care center

**DOI:** 10.1186/s42155-022-00316-z

**Published:** 2022-08-06

**Authors:** Philip Schuchardt, Lilla Kis, Alexey Goloubev, Edward Keshishian, Rahul Mhaskar, Glenn Hoots, Cliff Davis, Kamal Massis, Jamil Shaikh

**Affiliations:** 1 University of South Florida Department of Interventional Radiology, 2 Tampa General Circle, STC 6102, Tampa, FL 33606-3571 USA; 2 Morsani College of Medicine Office of Research, 12901 Bruce B. Downs Blvd., MDC061, Tampa, FL 33612-4799 USA

**Keywords:** IVC filter removal, Endobronchial forceps, Standard loop snare, IVC filter leg penetration, Tilted IVC filter

## Abstract

**Background:**

Inferior vena cava (IVC) filter retrieval is typically accomplished with standard snare technique. When this fails, more advanced techniques are necessary, especially when removal falls outside a 12-month window. Complications during filter retrieval depend heavily on technique, type of filter, and filter position. In this study we examined safety and efficacy of 536 filter retrievals at a tertiary care center and compared complication rates between standard snare and endobronchial forcep retrieval.

**Method:**

We reviewed 536 cases between August 2015 and August 2020, recording retrieval success rates, patient comorbidities, and complication rates at the time of removal.

**Results:**

Total overall retrieval success was 97.9% (525/536), and complications occurred in approximately 6.0% (32/536) of all cases. Success and complications with standard snare technique alone were 99.4% (345/347) and 1.7% (5 Grade I/II, 1 Grade III) and advanced forcep technique 98.8% (171/173) and 14.5% (22 Grade I/II, 2 Grade III, and 1 Grade IV), respectively. There was no significant difference between the technical success rates of the standard snare technique and forceps techniques (*p* = 0.60) despite a significantly longer dwell time in patients undergoing forceps retrieval (*p* < 0.001).

**Conclusion:**

To our knowledge, this is the largest cohort of forceps directed IVC filter retrieval present in the literature. Rates of successful endobronchial forceps and standard snare retrievals in this study are similar to previous reports. Although use of endobronchial forceps may be associated with higher complication rates, this is likely due to prolonged dwell times, filter tilt, and attempted removal of non retrievable filters. Overall, forceps-directed retrieval offers a safe, effective means of removal in difficult cases.

**Level of evidence:**

Level 3, Large Retrospective Study.

## Background

Inferior vena cava (IVC) filters, although excellent in the short term to prevent clinically significant pulmonary embolism (PE) in appropriate patients, are typically associated with a wide range of adverse events in the chronic setting, including increased risk of distal deep venous thrombosis (DVT) formation, filter migration, caval penetration, and filter fracture. Therefore, both the Food and Drug Administration (FDA) and Society of Interventional Radiology (SIR) recommend they be removed as soon as clinically indicated (Morales et al. 2013; Kaufman et al. 2020). Despite these recommendations, retrieval rates have been historically low. Mohapatra et al. published data of a large cohort of IVC filters and reported only 6.6% of 131,791 filters were successfully retrieved, while Everhart et al. in another study described retrieval rates of approximately 16% of prophylactic filters and 5.69% of therapeutic filters (Mohapatra et al. 2019; Everhart et al. 2017). Recent recognition of poor retrieval rates and the development of online filter registries and other standardized methods of patient follow up at select institutions have shown significantly improved filter removal rates, up to 66% (Schuchardt et al. 2019; Parsons et al. 2019; Minocha et al. 2010; Inagaki et al. 2016; Wang et al. 2016; Sutphin et al. 2015; Kallini et al. 2020).

Retrieval of IVC filters can be complex, and incorporation of advanced techniques is often necessary to maintain adequate removal rates. Filter retrieval has been shown to be successful in approximately 80–90% of cases using standard snare technique (Kuyumcu and Walker 2016). However, in cases when the filter hook cannot be engaged directly by a snare, alternative methods of retrieval are necessary (Kuyumcu and Walker 2016). Use of adjunctive and/or advanced removal techniques increase when the indwelling filter demonstrates high filter tilt, embedded struts, filter migration, fracture, endothelial overgrowth, and thrombus (Kuyumcu and Walker 2016; Stavropoulos et al. 2015). Recently, the utilization of endobronchial forceps has been widely accepted as a promising alternative technique for complex retrievals. Although a handful of published studies have demonstrated success using this technique ranging from 85 to 100% (Stavropoulos et al. 2015; Tavri et al. 2019; Chick et al. 2016; Avery et al. 2015), the majority of the literature is limited to case reports and case series (Nakashima et al. 2014; Cooper et al. 2018; White and Stavropoulos 2007; Johnston et al. 2014).

In this paper, we further investigate the use of endobronchial forceps as an alternative to the standard snare technique by evaluating overall filter removal success rates, patient outcomes, and complication profiles in 536 patients over a 5 year period. To our knowledge, our study is the largest cohort of forceps directed IVC filter retrieval present in the literature, providing additional evidence for the efficacy of this technique.

## Methods

Every patient who underwent IVC filter placement at our institution was placed in an internal registry for clinic follow-up. Eligibility for retrieval was periodically evaluated with chart review and primary physician consultation. When removal was indicated, patients were seen in clinic to discuss the benefits and risks associated with retrieval prior to scheduling the procedure. Additional referrals for filter removals were placed by outside institutions or inpatient consultations for complex retrievals, often involving permanent filters, intra-caval thrombus, or perforated struts in filters with prolonged dwell time. IVC filter retrieval was attempted if the filter was no longer indicated to prevent pulmonary embolism. This occurred when patients were able to tolerate anticoagulation, or when the patient was no longer deemed at increased risk for thrombus formation. Retrieval of permanent filters was often indicated in our cohort due to concurrent ileocaval thrombus distal to the filter or if it was no longer indicated and risk of keeping a permenant filter was felt to be clinically higher than removal (i.e. younger patients with high risk of thrombosis or malpositioned/fractured permenant filters). Retrieval with forceps was typically pursued directly in the setting of intracaval thrombus, dwell time of greater than 5 years, penetration into greater than 2 surrounding structures by perforated struts, and tilt greater than 10 degrees. In the absence of these findings, initial retrieval attempts were made with standard snare technique and if unsuccessful, conversion to forceps-directed retrieval was made intraprocedurally.

Standard snare technique was typically performed under moderate sedation, utilizing a combination of Fentanyl and Versed. In this technique, a loop snare was advanced through a 9F sheath nested in a 11F sheath toward the IVC filter hook. Once the hook was engaged by the snare, the filter was removed enbloc by resheathing the system. A final venogram was used to document caval injury after removal.

While conversion of standard snare technique to forceps-directed retrieval was often continued under moderate sedation, planned forceps-directed retrieval was performed under general anesthesia. Forceps directed retrieval required upsizing to an 18F sheath. Endobronchial forceps were then advanced through the sheath and used to engage the hook, often after removing overlying fibrin cap. Once the hook was engaged, removal was completed in a similar fashion to the standard snare technique, by resheathing the filter through the sheath.

Filter retrieval was also performed in the setting of chronic caval thrombosis in approximately 49 patients. The decision of overnight catheter-directed thrombolysis or mechanical thrombectomy was made by the attending physician depending upon the chronicitiy of the clot based upon imaging and symptoms and were often necessary before filter removal in these cases. After removal of clot in and around the filter, use of either standard snare technique or forceps-directed retrieval was performed at the discretion of the attending physician.

A single-center retrospective cohort study analysis of all consecutive filter retrievals between August 2015 and August 2020, was performed after institutional review board approval. The requirement for informed consent was waived due to the retrospective nature of the review. Overall, 536 IVC filter retrievals were attempted during this period. Medical records were reviewed to obtain patient age, sex, indication for filter placement, indication for retrieval, retrieval success rate, complications, the brand of filter, type of filter (permanent/temporary), dwell time, removal technique, access site, procedure time, fluoroscopic time, and volume of iodinated contrast administered. Filters were classified as temporary if they were designed for later retrieval, and they were classified as permanent if they were not designed specifically for later retrieval. This designation was made based on the brand of filter. All complications were classified using the Common Terminology Criteria for Adverse Events (CTCAE) version 4.03. Specifically, the category “vascular disorders- others, specify” was utilized for the categorization of complications.

### Statistical analysis

The data was stratified into standard snare and forcep retrieval subgroups based on removal technique. Any attempted retrievals that utilized forceps were characterized as a forceps directed retrieval, including those in which snare was used initially, concurrently, or after attempted forceps retrieval. The association of categorical variables was assessed by the chi-square or Fischer’s exact test to report the Mantel-Haenszel common odds ratios (OR) and 95% confidence intervals (95% CIs). The differences in the distribution of continuous variables (for example, dwell times) across the categorical variables (for example, Retrieval Technique) was investigated by the Mann-Whitney U test. *P* values less than 0.05 denoted statistical significance. The statistical analyses were conducted by SPSS software version 26 (IBM, Chicago, Illinois, USA).

## Results

Indications for insertion of IVC filters were separated into 7 categories and are listed in Table 1. Of the 536 filters, 31 filters were permanent, nonretrievable, while 505 were classified as retrievable. Ten complications occurred during the removal of permanent filters, while 22 occurred during the removal of retrievable filters, yielding complication rates of 32.3% and 4.4%, respectively. The complication rates associated with the removal of specific types of permanent filters and retrievable filters are summarized in Tables 2 and 3, respectively. At the time of removal, approximately 18 filters were already fractured. Three minor complications were observed during the retrieval of these fractured filters. Overall, 97.9% (525/536) of the filters were successfully removed, with a 6.0% complication rate (32/536). Filters that were not successfully removed were left in the IVC. The standard snare technique was attempted in 347 retrievals, isolated forceps were utilized in 105 cases, and a combination of both standard snare technique and forceps was used in 68 cases. Removal was not attempted in seven cases due to chronic thrombus where risks of removal outweighed benefits and/or recurrent pulmonary embolism. Success rates and graded complication rates for these different retrieval techniques are summarized in Tables 4 and 5.

**Table 1:** Indications for IVC filter placement

Indication for IVC filter placement	Number of filters
Contraindication to anticoagulation in the setting of VTE	187/536 (34.9%)
Placement in the pre/post operative period in setting of major surgery	134/536 (25.0%)
Pharmaco-mechanical thrombectomy for iliofemoral DVT	22/536 (4.1%)
Venous thromboembolism on therapeutic anticoagulation	20/536 (3.7%)
Extensive VTE	39/536 (7.3%)
Prophylactic placement (for example in trauma)	20/536 (3.7%)
Placement at outside hospital or with an unknown indication	114/536 (21.3%)

**Table 2:** Complications associated with attempted retrieval of permanent filters

Permanent Filter Complications			
Filter Type	Grade 1–2 Complications (Minor)	Grade 3–5 Complications (Major)	Complication Rate
Trapease	2	1	17.6% (3/17)
Venatech	1	0	50% (1/2)
Simon Nitinol	3	0	60% (3/5)
Greenfield	1	1	33.3% (2/6)
Birdsnest	1	0	100% (1/1)
Total	8	2	32.3% (10/31)

**Table 3:** Complications associated with attempted retrieval of retrievable filters

Retrievable Filter Complications			
Filter Type	Grade 1–2 Complications (Minor)	Grade 3–5 Complications (Major)	Complication Rate
Denali	2	0	1.3% (2/157)
Gunther Tulip	4	1	3.4% (5/147)
Optease	0	0	0% (0.8)
Bard G2	1	0	3.8% (1/26)
Bard Recovery	2	0	9.5% (2/21)
Eclipse	0	0	0% (0/5)
Celect	5	1	10.7% (6/56)
Option	3	0	4.1% (3/74)
Bard	3	0	42.9% (3/7)
Total	20	2	4.4% (22/505)

**Table 4:** IVC filter retrieval data stratified by retrieval technique. Success rate, complication rate, and dwell time are recorded

Technique	# Patients	Dwell time AVG (DAYS)	% SUCCESS	% Complications
Only SNARE	347	658	(345/347)99.4%	1.7% (6/347)
Only FORCEPS	105	2778	(104/105) 98.8%	13.3% (14/105)
Loop Snare + Forceps	68	1364	(67/68) 98.0%	16.2% (11/68)
OVERALL	536	1203	(525/536) 97.9%	6.0% (32/536)

**Table 5:** Graded complications for each retrieval technique. If multiple complications occurred during a single procedure, then the complication with the highest grade was recorded

Complications of IVC Filter Retrieval Based on Techniques of Removal						
	Grade 1	Grade 2	Grade 3	Grade 4	Grade 5	Total
Loop Snare	0.58% (2/347)	0.86% (3/347)	0.29% (1/347)	0% (0/347)	0% (0/347)	1.7% (6/347)
Forceps	7.6% (8/105)	2.9% (3/105)	1.9% (2/105)	0.95% (1/105)	0% (0/105)	13.3% (14/105)
Forceps + Loop Snare	7.4% (5/68)	8.8% (6/68)	0% (0/68)	0% (0/68)	0% (0/68)	16.2% (11/68)
Other Methods and Failed Filter Access	6.3% (1/16)	0% (0/16)	0% (0/16)	0% (0/16)	0% (0/16)	6.3% (1/16)
Total	3.0% (16/536)	2.2% (12/536)	0.56% (3/536)	0.19% (1/536)	0% (0/537)	6.0% (32/536)

The standard snare technique exhibited a success rate of 99.4% (345/347), with 1.7% (6/347) of patients experiencing complications. Among these filters, 9 were permanent, and 338 were retrievable. The majority of the complications in this cohort were low grade complications (Two Grade 1; 2/347, 0.58% and three Grade 2; 3/347, 0.86%). One patient experienced retroperitoneal bleeding after standard IVC filter removal (Grade 3; 1/347, 0.29%). A higher complication profile was seen in removal of permenant filters (2/5 complications, 40%). Filters removed with the standard snare technique had a mean dwell time of 658 days, ranging from 1 day to 5713 days. The median dwell time was 221 days.

Among all cases in which forceps retrieval was attempted, there was an overall 98.8% success rate (171/173). Filter retrieval utilizing only forceps had a 99.05% (104/105) success rate, though it also exhibited a higher complication profile compared to standard snare technique: 13.3% (14/105). Furthermore, higher grade complications were more prevalent with forceps retrieval (one Grade 4; 1/105, 0.95%; IVC rupture requiring intra-procedural placement of thoracic aortic stent graft, and two Grade 3; 2/105, 1.9%; retroperitoneal bleeding delaying discharge or requiring later hospitalization). Higher grade complications (grade 3 and 4) soley occurred during the removal of permanent filters. Eight low grade complications were observed (one Grade 1: 8/105, 7.6%; and three Grade 2 complications 3/105, 2.9%). The majority of low grade complications occurred with the removal of retrievabale filters (7/11, 63.6%). The mean dwell time was 2778 days, with a minimum dwell time of 1 day and a maximum of 10,075 days. The median dwell time was 2917 days.

Combined standard snare technique and forceps retrieval (failed snare removal converted to forceps or planned concurrent use of forceps and snare) was successful in 98.5% (67/68) of attempted removals, and complications occurred in 16.2% (11/68) of these procedures. Primarily, only low grade complications were observed with combined technique (five Grade 1 5/68, 7.4% and six Grade 2 6/68, 8.8%). Two of these minor complications (2/11, 18.2%) occurred during the attempted retrieval of a permanent filter. The mean dwell time was 1364 days (range: 4–7470 days). The median dwell time was 568 days. Of note, 51 of the 68 combined procedures involved conversion of failed standard snare retrieval to forceps retrieval.

As expected for a more complex procedure, radiation exposure was higher among patients undergoing forceps-directed retrieval. Standard snare technique resulted in an average of 7.4 minutes of fluoroscopy time, while forceps-directed retrieval (including simultaneous forceps and standard snare retrieval) resulted in an average fluoroscopy time of 18.2 minutes.

Any attempted filter removal that utilized forceps, regardless of concurrent technique, was classified as a forceps-directed retrieval for statistical analyses. Filters retrieved with forceps had a statistically significant longer dwell time (median: 1734 days; range(1–10,075 days) compared to filters removed with the standard snare technique (median: 221 days; range(1–5713 days), (*p* < 0.001). Despite this difference in dwell time, there was no statistically significant difference in retrieval rate between these two techniques (*p* = 0.60). However, complications were less likely in the standard snare technique group compared to the forceps retrieval group (OR:0.10; 95%CI:0.04–0.25), (*p* < 0.001).

## Discussion

VTE is often managed with anticoagulation therapy, but IVC filters provide necessary protection in the setting of DVT/PE in patients with contraindications to anticoagulation (Morales et al. 2013; Kaufman et al. 2020). While the use of IVC filters reduces the risk of PE, prolonged filter dwell time is associated with a range of potentially severe complications (Morales et al. 2013) and more difficult retrieval.

This center’s data was consistent with prior studies, demonstrating high retrieval success rates and low overall complication rates using both standard snare and advanced techniques (Kuyumcu and Walker 2016; Stavropoulos et al. 2015; Chen et al. 2019). Specifically, the rate of successful standard snare filter retrieval at this institution (99.4%) was significantly higher than the 80–90% success rates reported by Kuyumcu and Walker in the literature (Kuyumcu and Walker 2016). When accounting for failed standard snare retrieval attempts that were converted to forceps-directed retrieval, however, the success rate at this institution (87%, 345/397), was more in line with the rates reported in the literature.

Procedures that utilized the endobronchial forceps method of retrieval had an overall success rate of 99.05% (104/105). Similarly, Stavropoulos et al. demonstrated a success rate of 96% with endobronchial forceps filter retrieval (Stavropoulos et al. 2015). Furthermore, this institution’s results demonstrated a complication rate of 13.3% (14/105) with 3 major complications (classified as Grade 3 or higher), which was higher than the rate of 3.5% with one major complication reported by Stavropoulos et al. (Stavropoulos et al. 2015). Though Stavropoulos et al. had lower complication rates, the mean filter dwell time among their patients was 465 days (range 31–2976), while this institution demonstrated a mean dwell time of 2778 days (range 1–10,075 and median 2917). Prolonged dwell time is associated with an increased risk of filter fracture, migration, thrombosis formation in and around the filter, and strut penetration of surrounding structures (Desai et al. 2017). This type of embedded filter makes retrieval much more difficult and may result in higher complication rates.

Based on previous studies and this institution’s data, endobronchial forceps appear to provide a viable if not primary alternative to the standard snare technique in advanced filter retrievals. The success rates were similar- 99.4% (345/347) with standard snare, compared to 98.8% (171/173) with forceps (*p* = 0.60), despite the significantly longer dwell times of filters removed with forceps (*p* < 0.001).

This center’s complication rate was similar to published data with standard snare retrieval but higher for forceps retrieval. Longer dwell times and a higher proportion of permanent filters may explain this discrepancy. Standard snare technique had a much lower complication rate than forceps-directed filter retrieval. Additionally, major complications (defined as grade three or higher) were also more common with forceps removal, similar to published literature. Forceps retrieval of filters with longer dwell time, larger angles of tilt and concurrent caval thrombosis, if present, likely resulted in longer intraprocedural times, higher radiation doses, increased contrast doses and ultimately higher rates of complications. The higher complication profile of forceps mediated filter retrieval was therefore attributed to a combination of filter characteristics and concurrent medical comorbidities within this cohort, rather than the technique itself.

Overall, this data reaffirms that the use of advanced techniques, including endobronchial forceps filter removal, is equivocal in terms of success rates to standard snare retrieval. While this data provides good evidence for the safety and efficacy of IVC filter retrieval with endobronchial forceps, further data is necessary to assess the effect of confounding variables on complication rates. Additionally further studies are required to assess when forceps should be used as the primary method of retrieval as this is not yet known or studied.

## Conclusion

Endobronchial forceps and the standard snare technique had similar rates of successful IVC filter retrieval, despite the significantly higher dwell time seen in filters retrieved with forceps. Complication rates were higher in the forceps subgroup, though further studies will be needed to determine whether the complications are due to the increased dwell time or the removal technique itself**.**

## Data Availability

Reasonable requests for de-identified data may be accomadated by contacting corresponding author.

## Abbreviations

IVC: Inferior vena cava
PE: Pulmonary embolism
DVT: Deep vein thrombosis
VTE: Venous thromboembolism
FDA: Food and Drug Administration
SIR: Society of Interventional Radiology
CTCAE: Common terminology criteria for adverse events
OR: Odds ratio
CI: Confidence interval
